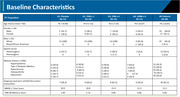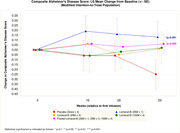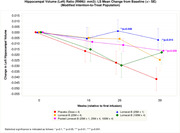# Results from a Phase 2a Proof‐of‐Concept Double‐Blind, Randomized, Placebo‐Controlled Trial of Lomecel‐B^TM^ in Mild Alzheimer’s Disease Dementia

**DOI:** 10.1002/alz.092295

**Published:** 2025-01-09

**Authors:** Kevin N. Ramdas, Nataliya Agafonova, Joshua M. Hare, Eric Naioti, Steven Kopcho, Jeffrey L. Cummings, Raul Carballosa, Paayal Patel, Mark Brody, Brad Herskowitz, Ana Fuquay, Savannah Rodriguez, Jeffrey Botbyl, Brian Rash, Anthony A. Oliva

**Affiliations:** ^1^ Longeveron Inc., Miami, FL USA; ^2^ Chambers‐Grundy Center for Transformative Neuroscience, Department of Brain Health, School of Integrated Health Sciences, University of Nevada Las Vegas, Las Vegas, NV USA; ^3^ First Excellent Research, Miami, FL USA; ^4^ Brain Matters Research, Delray Beach, FL USA; ^5^ Brainstorm Research, Miami, FL USA

## Abstract

**Background:**

Lomecel‐B is a novel cell‐based therapy with potential to demonstrate clinical benefit on Alzheimer’s disease (AD) and its progression. Here we present the results of a phase 2a proof‐of‐concept trial (n = 49) to further define the potential of Lomecel‐B in patients with mild AD dementia.

**Methods:**

This double‐blind, randomized, placebo‐controlled 45‐week trial (ClinicalTrials.gov: NCT05233774) enrolled patients (60‐85 yrs.) with mild AD dementia (MMSE score 18‐24); with evidence of amyloid on positron‐emission tomography (PET) and brain MRI consistent with AD. There were 4 study arms of Lomecel‐B intravenous infusion: 25 million (25M) cells once followed by 3 infusions of placebo (*N* = 13); 25 million (25M) cells (*N* = 13) or 100 million (100M) cells (*N* = 11) monthly for 4 months; 4 IV infusions of Placebo (*N* = 12). The primary endpoint was safety, secondary endpoint was change from baseline to Week 39 in Composite AD Score (CADS) that equally incorporated z‐scores of CDR‐SB, ADAS‐Cog‐13, ADCS‐ADL, and left hippocampal volume.

**Results:**

The trial achieved its primary endpoint of safety and tolerability (no infusion‐related reactions, ARIA or death). One SAE occurred within 30 days post‐infusion for each Lomecel‐B group with none for placebo. Lomecel‐B 25Mx1 demonstrated a trend toward slowing of disease progression relative to placebo on the CADS score (0.38 [‐0.06, 0.82], p = 0.091 (pre‐specified level of significance for CADS is p = 0.1)). While CADS declined by ‐0.25 [95% CI, ‐0.56, 0.07] in placebo, no decline occurred in the individual or pooled Lomecel‐B treatment groups. Regarding cognitive function, MoCA improved vs placebo (25Mx1: (4.90 [95% CI, 1.26, 8.55], p = 0.009) and (pooled: 3.74 [95% CI, 0.73, 6.75] p = 0.015). ADRQL and QOL‐AD were numerically improved in Lomecel‐B treatments; with significant improvement observed in the ADCS‐ADL for the 100M×1 (10.74 [95% CI, 0.50, 20.98] p = 0.040) and pooled group at Week 39 p = 0.047). Lomecel‐B slowed whole brain volume loss by 49% (100Mx4 (8.31 [95% CI, 0.12, 16.50] p = 0.034) with significant preservation of left hippocampal volume (pooled: 0.0253 [95% CI, 0.0015, 0.0492] p = 0.038), Week 39 compared to placebo.

**Conclusion:**

Together, the study achieved proof‐of‐concept in a small sample size. Accordingly, these findings support further development of Lomecel‐B in a larger dose‐finding study.